# Severe Tracheobronchomalacia after Prolonged Intubation of Multitrauma Patient

**DOI:** 10.1155/2011/627012

**Published:** 2011-08-24

**Authors:** V. G. Sams, C. M. Lawson, A. B. Shibli, D. A. Taylor, P. R. Branca

**Affiliations:** ^1^Department of Surgery, University of Tennessee Medical Center, Knoxville, TN, USA; ^2^Division of Trauma Critical Care, Department of Surgery, University of Tennessee Medical Center, Knoxville, TN, USA; ^3^Section of Critical Care Medicine, Department of Internal Medicine, University of Tennessee Medical Center, Knoxville, TN, USA

## Abstract

Tracheobronchomalacia is a condition with significant morbidity with many etiologies including iatrogenic ones and should be considered in critically ill ventilated trauma patients. We present a case of a multitrauma patient who had difficulty weaning from the ventilator after prolonged intubation followed by tracheostomy tube placement. We describe her presentation, diagnosis, and management provide and as well a discussion of the condition.

## 1. Introduction

Tracheobronchomalacia (TBM) is defined as the loss of cartilaginous support of the trachea or major bronchi, leading to complete or near-complete collapse of the airway lumen during respiration. When this is isolated to segments of the trachea, the cartilages are either destroyed such as in intubation injury or softened by chronic extrinsic compression such as with congenital vascular malformation or intrathoracic goiters [1].

## 2. Case Report

The patient was a 57-year-old female who presented to the trauma bay of a university trauma center via intrafacility transfer after a motor vehicle collision in which her vehicle rolled down an embankment. She was hemodynamically stable and with a Glascow Coma Score of 15. Computed tomography of her chest abdomen and pelvis revealed an aortic injury consistent with transection and left hemothorax with associated atelectasis. Her blood pressure was stable, and her hemoglobin and hematocrit were within normal limits. She had several comorbidities which included chronic obstructive pulmonary disease, diabetes mellitus, hepatitis C, hypertension, history of thyroid cancer status post thyroidectomy, and asthma. 

### 2.1. Findings

She was admitted to the surgical critical care unit for strict blood pressure control and went to the endovascular suite with vascular surgery the following morning for a thoracic endovascular graft repair of the aortic injury. The case resulted in a midline laparotomy after deployment of the graft, lengthy lysis of adhesions, injury to the left gonadal vein resulting in one liter of blood loss, and finally retroperitoneal dissection and exposure of the infrarenal aorta and iliac arteries. Due to the extensive calcifications of the iliac vessels, the aorta was cannulated and the stent graft was deployed. Her abdomen was closed, and she was returned to the critical care unit.

The patient's course in the critical care unit was long and complicated to include acute respiratory distress syndrome and prolonged mechanical ventilation. Due to high oxygen and positive end-expiratory pressure requirements, patient did not undergo a tracheostomy until hospital day number twenty-four. She subsequently had a PEG tube inserted a few weeks later. We continued to attempt to wean the patient from mechanical ventilation. Her Glasgow Coma Score improved significantly over the following weeks to the point she was alert and able to follow commands, attempting to verbalize. However, when patient was placed on tracheostomy collar trials, she became very anxious and tachypneic. This was felt initially to be due to anxiety. After several days of similar symptoms despite anxiolytics, patient developed a wheeze. She had no changes in her chest radiograph. We decided at that time to perform a flexible bronchoscopy and found tracheal collapse distal to the tracheostomy tube.

### 2.2. Diagnosis and Management

We ordered a dynamic computed tomography scan of the chest to evaluate the extent of the tracheomalacia. Imaging revealed significant collapse of the distal trachea and main stem bronchi. It was decided to take her for video bronchoscope, rigid bronchoscopy, Dynamic Y-stent placement, and exchange of tracheostomy tube with a regular Shiley size 8 tracheotomy tube (Figure 3).

Videobronchoscopy was performed through the distal XLT (Extended Length) tracheostomy tube, and severe tracheobronchomalacia was noticed. The patient had almost complete collapse of the trachea distal to the tracheostomy tube and almost complete collapse of the left mainstem bronchus. Distal XLT tracheostomy tube was exchanged to regular Shiley size 8 tracheostomy tube. Videobronchoscopy was performed through the newly placed Shiley tracheostomy and measurements for the Y-stent taken. Rigid bronchoscopy was then done to ascertain that patient was rigidly intubatable for the deployment of dynamic Y-stent (Figure 2). After successful rigid bronchoscopy Y-stent was deployed under fluoroscopic guidance. The Y-stent limbs were well seated in the trachea left main stem bronchus and right main stem bronchus. 

Postoperatively patient did well and was able to be weaned from mechanical ventilation. At three-month followup, patient is doing well with tracheostomy and stent in place. The plan is to follow the patient for stent and/or tracheostomy removal or replacement.

## 3. Discussion

Recurrent intubation and duration of intubation may directly predispose to TBM. They are the most common cause of adult acquired TBM [2]. Acquired diffuse tracheobronchial collapse is observed with chronic obstructive pulmonary disease (COPD) and is believed to represent the chronic inflammatory response of larger airways to tobacco smoke. Other less common causes of tracheal collapse include congenital tracheobronchomegaly, Mounier-Kuhn disease, and relapsing polychondritis [1]. Large thyroid goiters have often been implicated in cases of tracheomalacia. In a study in India, an area of endemic goiter, review of 900 thyroidectomies for goiter revealed a 1.9% incidence of airway collapse related to tracheomalacia [3]. Intrathoracic collapse occurs during expiration, whereas extrathoracic malacia results in inspiratory collapse. 

**Figure 1:**
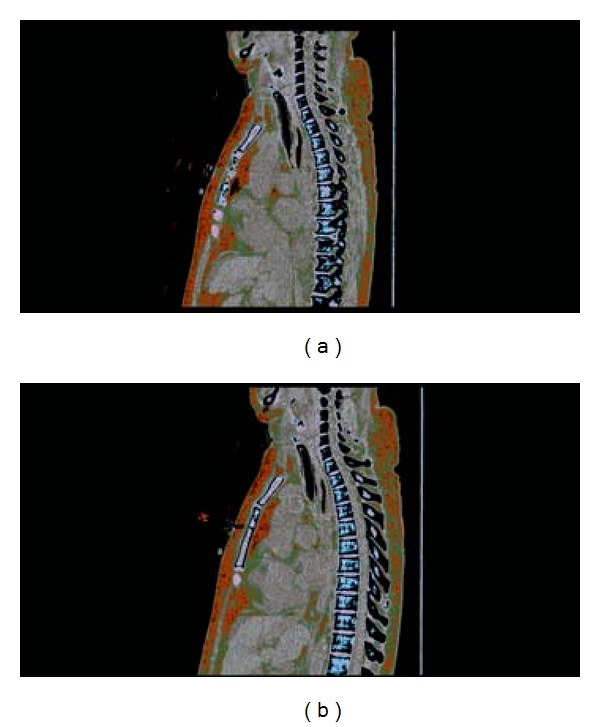
CT thorax, inspiration (a) and expiration (b), the figure tracheomalacia protocol; demonstrates near complete collapse of the distal trachea during expiration.

**Figure 2:**
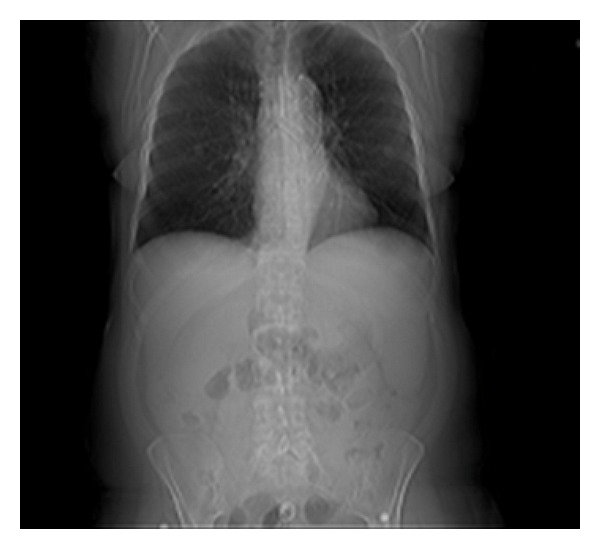
Portable CXR after stent deployment.

**Figure 3:**
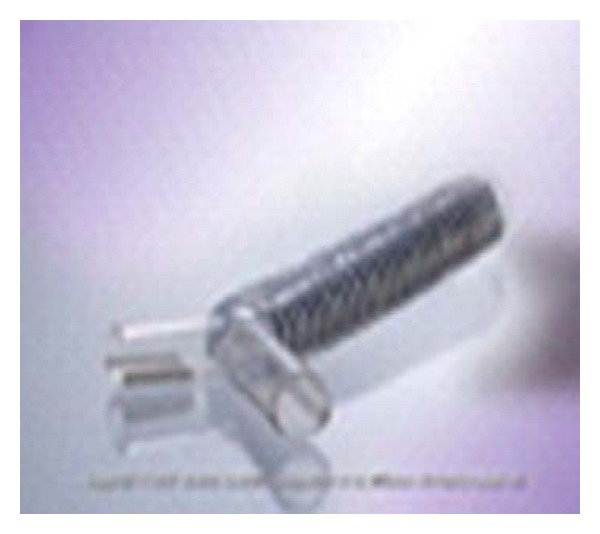
Dynamic Y-stent.

The diagnosis is made either by multidetection computed tomography (MDCT) or by fiber optic bronchoscopy. Characteristic of acquired tracheomalacia in patients with COPD is complete anteroposterior collapse with apposition of cartilage and membranous portion during expiration; inspiration enlarges the lumen (Figure 1) [1]. Incidence of tracheobronchomalacia, defined as being greater than 50% decrease in lumen diameter at the end expiratory phase with paired inspiratory—expiratory MDCT using a low-dose technique, has been reported to be 7–15% [4, 5] and up to 50% of all types to include tracheal, tracheobronchial, and bronchial malacia in patients with COPD [6].

## 4. Conclusion

Patients with diffuse tracheobronchomalacia are selected for surgical treatment if the severity of malacia exceeds that of small airway disease; this is usually the case when their collapse is complete and extends over the entire trachea. Temporary tracheal stenting can be beneficial in select patients [1]. The dynamic features of TBM are quite different from static causes of central airway obstruction. Constant change in size and shape of the airway predisposes to stent migration and fracture. This makes appropriate selection of the type and size of airwaY-stent difficult [2]. Metallic stents are an option for patients with large airway compromise secondary to benign airway diseases for whom other medical comorbidities contraindicate surgery. Once deployed, they are difficult to remove, are associated with significant complications, and require prospective bronchoscopic surveillance and often further therapeutic intervention [7]. Surgical therapy consists of restoring the convex cartilage horseshoe shape by reefing and supporting the membranous wall with polypropylene mesh [1].
